# Conceptual inflation, communication and understanding, and collective attention

**DOI:** 10.1007/s11098-025-02374-0

**Published:** 2025-07-14

**Authors:** Eve Kitsik

**Affiliations:** Department of Philosophy, University of Vienna, Universitätsstraße 7, 1010 Vienna, Austria

**Keywords:** Conceptual inflation, Ethics of attention, Collective attention, Language change and attention, Language change and communication, Language change and understanding

## Abstract

Some sociologists, psychologists, and philosophers, among others, have expressed worries about the inflation of concepts related to negative experiences, harm, or injustice (for example, the concepts of racism, sexual harassment, and human rights). Others welcome and contribute to the linguistic changes. What is at stake in these disagreements? In this paper, I first give an account of what conceptual inflation, in one important sense, is: change in linguistic practices that makes it easier to indicate a problem of a certain category. Then, I argue—building on work by Shen-yi Liao and Nat Hansen—that such conceptual inflation of problems neither significantly limits nor enhances our ability to communicate about the relevant sub-problems (such as workplace harassment and street harassment) and to recognize the similarities and differences between them. This is because our linguistic resources are flexible, and we are capable of creatively using and developing those resources. There is, however, another way of making sense of the disagreements. The issue at stake could be the allocation of collective attentional resources to (alleged) problems. There are plausible mechanisms whereby how we use the terms in question (such as “sexual harassment”) influences that allocation.

## Introduction

The “inflation” or “creep” of concepts related to negative experiences, harm, injustice, or disorder (for example, the concepts of racism, sexism, bullying, sexual harassment, violence, safety, human rights, and addiction) has recently been discussed by academics (Haslam, 2016; Campbell & Manning, 2018, pp. 86–97; Case, 2019; Tasioulas, 2021) and in the media (Friedersdorf, 2016; Williams, 2017). It is not quite clear what conceptual inflation is, nor what the problem with it is supposed to be. A prevalent concern is that these linguistic changes threaten communication and understanding. Some, however, welcome the conceptual expansion as a progressive development (Cikara, 2016; Hardimon, 2019; Theilen, 2021). In this paper, I offer a perspective that makes sense of many disagreements between the pro- and anti-inflation sides. That perspective brings the discussion on conceptual inflation together with a recently flourishing field in philosophy: the ethics of attention.

I will begin (in Sect. 2) by giving a descriptive account of one important kind of conceptual inflation: the conceptual inflation of problems, which happens when it becomes more common and more generally accepted to use a term (for example: “sexual harassment”, “human rights”) on more sorts of occasion to indicate a problem of a certain category (for example: sexual harassment, human rights violations). Then (in Sect. 3), I will consider and reject a focus on whether such conceptual inflation undermines or enhances communication about the relevant sub-problems, such as workplace harassment and street harassment, and our understanding of the similarities and differences between these sub-problems. I will argue (in Sect. 4) that there is something else at stake in many debates between the anti- and pro-inflation sides: the distribution of collective attentional resources. How we use the terms in question (such as “sexual harassment) affects what the community treats as prioritized problems in allocating resources such as funding, time, and space. I will further argue (in Sect. 5) that this perspective helps us to move forward with the disagreements in a rational manner by identifying and addressing the relevant empirical and normative issues.

## The conceptual inflation of problems

Let us first look at some examples of alleged conceptual inflation. Sociologists Miles and Brown have argued that the concept of racism “has been redefined to refer to a wider range of phenomena” (Miles & Brown, 2003, p. 58). After the second world war, “racism” was primarily associated with belief in the natural superiority or inferiority of certain races but then came to be increasingly used for other phenomena, such as “all processes that, intentionally or otherwise, result in the continued exclusion of a subordinate group” (Miles & Brown, 2003, p. 66). More recently, Haslam’s discussion of “concept creep” has been influential in psychology (Haslam, 2016; Haslam et al*.*, 2020). Haslam is concerned with concepts that “refer to the negative aspects of human experience and behavior” (Haslam, 2016, p. 1) in the discipline of psychology, for example, the concepts of abuse, bullying, trauma, mental disorder, addiction, and prejudice. “Abuse”, Haslam suggests, has broadened to include emotional abuse and neglect in addition to physical and sexual abuse, and from adults abusing children to behaviors exchanged between adults (Haslam, 2016, p. 3). “Bullying”, for another example, has spread from schools to the workplace, the repetition requirement has been loosened, intention is no longer required, and the victim’s perception of events has become decisive (Haslam, 2016, pp. 4–5).

In philosophy, debates have focused on the inflation of the concept of racism (e.g., Blum, 2002; Cabezas, 2024; Hardimon, 2019); there has not been much systematic discussion of the broader phenomenon. Hansen and Liao’s work on the topic (Liao & Hansen, 2023; Hansen and Liao, forthcoming) is notable but still a limited to a narrow range of examples, among which “racism” is again the central one. Case (2019) and Tasioulas (2021) discuss the broader phenomenon but do so in a perfunctory manner. Case, whose examples include concepts such as gaslighting, violence, colonialism, sexism and racism, defines “concept inflation” as “what occurs when speakers loosen the usage of an emotionally impactful word in order to manipulate an audience” (Case, 2019). Case thus focuses on instances where emotionally impactful terms are used manipulatively in not-yet-standard ways, leaving aside unintended or non-manipulative linguistic changes. Tasioulas (2021) observes that certain politically significant concepts, such as those of democracy, health, and human rights, are being enriched with further ideas that were originally not part of the concept. For example, such conceptual inflation occurs when “health” is increasingly taken to require complete well-being rather than mere absence of disease or infirmity; when human rights are taken to include not just rights such that “it’s seldom if ever justified to override them” but also other important objectives (like internet access); and when democracy is equated with liberal democracy. The concepts are inflated, according to Tasioulas, in the sense that our understanding of health, human rights, or democracy becomes unnecessarily “bloated” and we “lose sight” of the distinct ideas originally conveyed by the terms.

In order to make sense of the debates about conceptual inflation, we first need to set the phenomenon at issue apart from related but distinct ones, such as patterns of using a word in a non-literal sense (for example, the word “literally” itself has come to be used in a non-literal intensifying sense, as in “It was so funny, I literally peed my pants”) or uncontested politically insignificant semantic change (for example, the meaning of “awesome” changing from “inspiring awe or dread” to “impressive, very good”). The following characterization covers many cases that have been discussed under the label “conceptual inflation” and isolates a phenomenon that is specific enough for a sensible discussion on how to assess it.

*Conceptual inflation of problems*: The practices of using a term change so that it becomes easier to indicate a problem of a certain category by using that term. In other words: the conditions for indicating a problem of a certain category are relaxed. This means, more precisely, that people are inclined to (1) *use* the term to indicate a problem of a certain category in a wider variety of cases; and (2) *accept* it as appropriate when others use the term to indicate a problem in a wider variety of cases.

Often, the relevant term itself denotes the problem that it is used to indicate. “Sexual harassment”, “bullying”, and “gaslighting”, for example, denote the problems of sexual harassment, bullying, and gaslighting. It becomes easier to use the term “sexual harassment” to indicate a sexual harassment problem when it becomes commonplace to use the term when talking about behaviors of decreasing severity and about those occurring, say, on the streets in addition to those occurring at the workplace, and such widening usage is also increasingly accepted as appropriate. Those worrying about conceptual inflation sometimes also discuss the inflation of terms that do not themselves denote problems, such as “democracy”, “human rights”, and “health” (examples from Tasioulas, 2019), or “safety” and “empathy” (examples from Haslam et al*.*, 2020, pp. 257–8). My account explains how conceptual inflation can also occur for such terms: the practices of using these terms can (and arguably do) also change so that it becomes easier to denote problems of certain categories, such as human rights violations or threats to safety. For example, when safety is increasingly taken to require not just physical safety but also feeling psychologically secure, it becomes easier to indicate threats to students’ safety on campus. As Campbell and Manning put it: “What constitutes safety is defined more narrowly, while threats to safety are defined much more broadly” (Campbell & Manning, 2018, p. 89). What really inflate (widen), in all cases, are the relevant categories of problems, which come to include more sorts of sub-problems. The categories widen because the way we use certain terms (such as “human rights” or “sexual harassment”) changes. So, conceptual inflation (of problems) is the widening of problem categories through conceptual change, where conceptual change means change in our practices of using the relevant terms.

Conceptual inflation is often glossed as a kind of *meaning* change. For example, Haslam writes that the relevant concepts “have undergone semantic shifts” and “have expanded their meanings so that they now encompass a much broader range of phenomena than before” (Haslam, 2016, p. 1). On my account, conceptual inflation involves changes in linguistic practices and not necessarily changes in meaning. The account is therefore compatible with different views on what meanings are, how they relate to linguistic practices, and what it takes for meanings to change. This descriptive account is also evaluatively neutral, in contrast to some other definitions of conceptual inflation. (For example, according to Case’s (2019) definition quoted above, speakers who engage in conceptual inflation are trying to “manipulate” their audience.)

Conceptual inflation, on this characterization, does not necessarily emerge from the political left. Pro-inflation attitudes are often associated with progressive left-wing politics (Campbell & Manning, 2018; Haidt, 2016; Haslam, 2016). The comparatively conservative side, however, appears to be pro-inflation regarding terms like “censorship”, “silencing”, “freedom of speech”, or “cancelling”—contributing to and welcoming changes in linguistic practices that make it easier to raise worries about censorship, threats to freedom of speech, and so on, thus widening these categories of problems. Contemporary progressives, on the other hand, tend to be against inflating these problem categories. For example, Yap and Ichikawa mention “the comically lax way in which talk of ‘cancellation’ is thrown around today” (Yap & Ichikawa, 2024, p. 267) and describe Kathleen Stock’s complaints about being “silenced” by the critical open letter about her as “catastrophizing exaggeration” (p. 271).

My account does not include everything that some might want to call “conceptual inflation”. For example, it does not include changes in how we use terms like “awesome” or “fantastic”. While there may be interesting similarities between, say, changes in the usage of “awesome” and the usage of “sexual harassment”, there are also advantages to the narrower focus on the conceptual inflation of problems. The latter has special characteristics. In particular: when usage changes so that it becomes easier to indicate certain problems, political views apparently play a role in whether one is against or in favor of the trend. Our opinions on such changes are not just a matter of whether we are generally pernickety regarding “correct” language use. Some progressives, for example, are pro-inflation about “sexual harassment” and (threats to) “safety” but anti-inflation about “cancellation” and (infringements on) “freedom of speech”, and this has something to do with their politics. There seems to be something more, or different, at stake here than in other sorts of debates about non-literal or widening usage. And what is at stake here is precisely the topic of this paper.

The focus on the conceptual inflation of problems is also more or less in line with how, for example, Haslam as well as Liao and Hansen have delineated the phenomenon. Haslam is concerned with “[c]oncepts that refer to the negative aspects of human experience and behavior”, also describing these as “concepts of harm and pathology” (Haslam, 2016, p. 1). Liao and Hansen focus on “expressions condemning oppression” (such as “racism”, “sexism”, “homophobia”, “colonialism”), which “refer to and connect a particular instance—such as a person, an event, or an institution—to broader patterns of oppression and do so with the force of moral condemnation” (Liao & Hansen, 2023, p. 74). “Problem” is meant to be an umbrella term that covers the various categories of negative, oppressive, harmful, abnormal, illegitimate (and so on) phenomena that widen when our usage of the terms in question changes.

“Conceptual inflation”, in this sense, does not cover linguistic widening that has nothing to do with the widening of problem categories. But it does cover cases where the community’s understanding of a problem category expands so that many people include under this category phenomena that, as many other insist, are not problems at all. For example, suppose that many people start using “safety” so that exposing students to offensive views counts as a threat to their safety, while others think that such exposure is not problematic and is even desirable. This is conceptual inflation, on my account—so, the account is not restricted to cases where everyone agrees that the phenomena newly included under the problem category are genuine problems, albeit perhaps less serious or importantly different ones.

The worry about the conceptual inflation of problems, in broad strokes, is that it becomes *too* easy to use a term to indicate a problem of a certain category. Others, by contrast, consider it a good thing that it becomes easier (in certain ways) to point out problems of certain categories. How to understand and approach the disagreement between these anti-inflation and pro-inflation sides is the topic of this paper.

## The worries about communication and understanding

A possible view that I will simply set aside is that there are deep metaphysical truths about what is really racism, sexual harassment, violence, and so on, and that we should figure these truths out and let them inform how we use terms like “racism”, “sexual harassment”, and “violence”. According to such a view, the debate between the anti- and pro-inflation sides is ultimately a metaphysical one—for example, about whether sexual harassment can *really* take place on the streets. That metaphysical approach offers intolerably little guidance on establishing the metaphysical facts and, in any case, it remains unclear why our linguistic practices should follow those facts. Furthermore, I will not be concerned with assessing the broader cultural changes (such as increased sensitivity, the rise of progressivism, or the emergence of a culture where it pays to be a victim (Campbell & Manning, 2018)) that allegedly cause much of the conceptual inflation of problems. I will only be concerned with assessing the *effects* of the inflation itself, from a broadly pragmatic, epistemic, and ethical perspective.

The anti-inflation side’s worries often revolve around threats to communication and understanding. Case (2019), for example, expresses a worry about communication related to the inflation of “gaslighting”: “Because ‘gaslighting’ is a label for a kind of bad behavior that has no other convenient designation, inflating this word’s meaning hampers our ability to communicate.” Tasioulas (2021) expresses a worry about understanding when he writes that the inflation of concepts like human rights, democracy, and health is “a source of intellectual confusion” and makes us “lose sight of the distinctive idea conveyed by a given concept through its immersion in a sea of many other quite separate ideas”. Let us look at these anti-inflation arguments, and the corresponding pro-inflation arguments, in turn.

In the light of my descriptive account of the conceptual inflation of problems, the worry about communication could be that it becomes exceedingly difficult to clearly indicate the problems that the terms were previously used to indicate; confusion and misunderstandings thus proliferate. For example, one might want to point out a problem with health in the narrower sense of “health” (roughly, absence of disease or infirmity) when one utters, “The state of people’s health in this country is unsatisfactory”. The audience, however, aware that “health” now encompasses total well-being, might be confused as to what problem precisely is at issue and what could be done to address it. Or, for another example, an employee might want to express the idea that the company is “racist” in the narrow sense that the individuals in it harbor overtly discriminatory and hostile attitudes; but the manager receiving the complaint might take it to concern indirect structural discrimination or subtle insensitivity that abounds in enterprise and culture generally. So, miscommunication results from conceptual inflation. A natural response from the pro-inflation side is that conceptual inflation *enhances* communication about the problems that we previously did not have well-known terms for. For example, when unwanted, disrespectful advances on the street or in public transportation were not recognized as sexual harassment, it was more difficult for the targets of these behaviors to express what happened and to complain about it.

The problem with these arguments is that they underestimate the flexibility of our linguistic resources as well as our creativity in using these resources and inventing new ones. Liao and Hansen (2023) make an important point in this regard: our flexible linguistic resources (including, but not limited to, degree modifiers like “slightly” and “extremely”) allow us to efficiently communicate the more specific ideas that we have in mind, even when the umbrella categories (like racism or sexism) are rather broadly understood. As Liao and Hansen put it, “English speakers have a rich linguistic repertoire for qualifying the degree to which and dimensions along which something is racist, sexist, homophobic, and so on” (Liao & Hansen, 2023, p. 72). This rich linguistic repertoire helps us to get our messages across even when, say, racism or sexism are increasingly understood in a broader way. For example, our language has resources for specifying what kind of health problem is at issue or what kind of (or degree of) racism is present in the company; and we can trust speakers to be creative enough to draw on these resources to get their intended message across.

The same point applies to the corresponding pro-inflation argument, according to which inflation *enhances* communication because it gives us better resources for communicating about problems that used to be excluded from categories like “sexual harassment” or “bullying”. Arguably, we already had sufficient resources to communicate about those problems—to get across what happened or is happening and what was or is wrong about it. We could talk about “disempowering and disrespectful unwanted advances on the streets” (instead of “sexual harassment on the streets”) or “demeaning, hostile behavior by superiors and colleagues at the workplace” (instead of “workplace bullying”). Indeed, perhaps these more elaborate descriptions get the issue across more effectively. And even where words—or sufficiently snappy words—really are lacking, speakers’ creativity comes to aid. We can, usually through collective efforts, come up with and secure uptake for new snappy words, instead of incorporating all related or similar problems that we need to talk about under the same existing category.

What about arguments that invoke the notion of *understanding*, rather than that of communication? The anti-inflation case here is that when we gather distinct problems under a single category, it is harder to grasp the important differences between the problems. For example, Maitra is concerned about the distortions introduced by the expanding notions of rape and sexual harassment. Maitra acknowledges that we can make our experiences and their normative status more communicatively intelligible by assimilating the experiences to those “whose relevant normative properties are already sufficiently familiar” (Maitra, 2018, p. 353). Maitra argues, however, that in the course of such assimilation, we also occlude important differences between the experiences. For instance, the concept of rape that includes statutory rape “neither collects together behavior that constitutes similar enough wrongs, nor behavior that merits similar treatment” (Maitra, 2018, p. 358). Among other reasons, this is because statutory rape in many cases includes consent “on any reasonable understanding of that notion” (ibid., p. 356). Maitra also asks whether the concept of sexual harassment that includes, say, *quid pro quo* as well as hostile environment harassment might gather together normatively different matters in a way that undermines our understanding of these wrongs and the appropriate responses. Essentially the same point about the concept of sexual harassment has often been raised in the public debate on the #MeToo movement. For example, Joanna Williams (2017) writes that a rather narrow concept of sexual harassment would allow us to “better separate trivial irritations from criminal behaviours”.

The corresponding pro-inflation argument is that the broader categories of problems help us to appreciate the important *similarities* between the phenomena. For example, extending the category of sexual harassment from the workplace to the streets helps us to see what the apparently different behaviors occurring in different contexts have in common and how they combine to form a broader phenomenon that threatens women’s equal participation in society, due to the accumulation of demeaning, objectifying, and disempowering experiences (Crouch, 2009). We would not understand the full extent of the problem if we limited the concept to contexts such as the workplace or schools, one might say. For another example: Haslam mentions, as motivation for extending the term “addiction” beyond substance addiction, that “certain compulsive behaviors overlap substantially with substance addictions in their phenomenology, neurobiology, natural history, personality correlates, and response to treatment” (Haslam, 2016, p. 8). Perhaps, then, in order to better grasp these similarities, we ought to extend the term “addiction” to these compulsive behaviors.

Both the anti-inflation and the pro-inflation arguments here involve an overly strong and unmotivated variety of linguistic determinism. Our language does not constrain our ability to grasp the similarities and differences between phenomena in the manner and to the extent assumed in these arguments. As Barber and Stainton point out:

Spanish speakers aren’t confused about the difference between toes and fingers just because they use ‘*dedos*’ for both. Conversely, English-speaking biology students aren’t rendered less capable of absorbing information about the structural parallels between toes and fingers by their use of different words for each. (Barber & Stainton, 2021, p. 58)

The “direct and inflexible influence of the lexicon on psychology”, they conclude, is “overhyped” (ibid.). Spanish speakers do not stick their toes in the food or put socks on their hands. So, it is unclear why we should be blinded to the differences between, for example, one-time street harassment and repeated workplace harassment when we group both under “sexual harassment”. Conversely, we need not indicate similar problems using the same term, in order to see the similarities. For example, we can grasp the similarities between workplace harassment and demeaning advances in public places, and that they are part of a broader cultural tendency and contribute to gender inequality, even if we use different terms for these problematic phenomena.

More precisely, the problem with these anti- and pro-inflation arguments is that they overestimate how much our linguistic resources influence what perspectives we can adopt on the phenomena in question. As Sliwa (2024) argues, what really matters for making sense of the normative features of a situation is the aptness of our perspective. Sliwa initially describes how classifying an experience as “rape” opens up a certain perspective on the experience, involving “a holistic shift of outlook”, which “affects what stands out to her and how she sees certain features as mattering: her saying *no*, the fact that she was too drunk for consent, the fact that she was followed” (Sliwa, 2024, p. 121). Sliwa seems to agree, however, that while certain familiar words might be useful for evoking an apt perspective, it is ultimately not the application of the right word that matters for properly making sense of the situation; it is having the apt perspective. Many linguistic routes lead to an apt perspective. So, we can identify certain features of the situation as salient, foreground certain values, desires, and commitments, draw certain inferences, visualize certain things, and have a certain affect (in sum: have a certain perspective), without applying a certain term.

For example, Sliwa writes that “by settling on the perspective of rape, Kristen’s project could be successful even if she remains uncertain about whether what happened to her should be categorised in this way [as rape]… in virtue of attuning her to a range of features about her situation, which may allow her to locate her experience in a family of wrongs (sexual assault, rape)” (Sliwa, 2024, p. 130). Drawing on this: When we do not take the term “sexual harassment” to include demeaning advances in public places, we will not be led by the application of that term to view these advances through a certain lens, from a certain apt perspective. But we are still able to take up an apt perspective, for example, by thinking of these behaviors as *relevantly similar* to sexual harassment, or via some other illuminating description. Likewise, even when we think of “sexual harassment” in a broad way, we can still adopt more nuanced, differentiating normative perspectives on the various phenomena that fall under it, using further linguistic resources to characterize the situation more fully.

To sum up this section: Our linguistic resources are flexible enough and we as language users are capable enough to express ourselves clearly, even when the resources for expressing ourselves change. Furthermore, the flexibility of language and our own mental capacities also allow us to take nuanced, apt perspectives on problematic phenomena, regardless of whether we understand problems like sexual harassment, bullying, and so on in a broader or narrower way. Thus, it does not make much sense to debate whether we should resist or encourage conceptual inflation, in so far as we are merely concerned with the effect of conceptual inflation on our ability to communicate about and understand the relevant problems. At least, it ought to be made clearer how exactly conceptual inflation is supposed to threaten or enhance communication and understanding. I will not pursue this route further myself, although perhaps my alternative account can be understood in these terms as well. (I will return to this point.)

## The attention allocation account

I suggest, then, that there is something else at stake in the disagreements on conceptual inflation: namely, how the community should distribute its attentional resources between various (alleged or genuine) problems. Something in the vicinity of this idea has been expressed by others, in more and less explicit ways; I will make the idea more precise.

Let us begin with examples of this idea or something in the vicinity. Shelby suggests that we “define the scope of ‘racism’ by focusing on those race-related ills that have the greatest consequences for the liberty, material life prospects, and self-respect of individuals” and “shift our critical eye away from identifying racist individuals and toward understanding the subtle dynamics of institutional racism” (Shelby, 2014, p. 61). Miles and Brown have earlier summarized this sort of rationale as follows: “*x* is what matters, therefore *x* is racism” (Miles & Brown, 2003, p. 70). Haslam et al*.* point out that concept creep “can motivate interventions aimed at preventing or reducing the harms associated with the newly categorised behaviours”; for example, recognizing gambling and internet “addictions” has motivated research on treating these conditions (Haslam et al*.*, 2020, p. 278). These suggestions, however, leave us in dire need of *mechanisms.* How does extending the term “racism” to institutional racism help to “shift our critical eye”? How does classifying compulsive gambling as an “addiction” motivate research on treating compulsive gambling? I will first briefly explain what (collective) attention, in the relevant sense, is, before turning to the mechanisms whereby the conceptual inflation of problems redistributes collective attentional resources.

The most uncontroversial thing to say about attention is that it means mental prioritization, either of representations or of worldly phenomena. For example, according to Jennings, attention is “the prioritization of mental and/or neural processing on the basis of the subject’s current interests” (Jennings, 2020, p. 107). According to Mole (2011), “A theory of attention is an attempt … to explain the *selectivity* of our mental engagement with the world” (Mole, 2011, pp. 3–4). (For Mole, selectively engaging with the world means that our relevant cognitive resources are employed fully in the service of the engagement—there is “cognitive unison”.) Watzl characterizes attention as the activity of regulating priority structures—structuring the mind into centre and periphery, foreground and background. According to Watzl, “the central function of attention is to organize, integrate, and coordinate the various parts of the mind by means of prioritization” (Watzl, 2017, p. 108). Wu focuses on the role of attention in guiding action, that is, in selecting among the many things in the world something to act on and the action to perform with it: “Attention is a selective mental orientation for the sake of guiding action” (Wu, 2023, p. 66).

We need not settle on a theory of attention to make sense of what it means for a community to prioritize some problems over others. Collective prioritization, as I will explain further below, arises from individual mental prioritization, and means the allocation of collective attentional resources—funding, time, space, mental effort, and so on. But Wu’s (2023) account of attention as selection for action is useful for our purposes. How we use terms like “sexual harassment” or “human rights” influences what problems we select for acting on (addressing), first as individuals, and thereby as communities. Perhaps it is possible to prioritize something in one’s mind without selecting it for action in any sense. For present purposes, however, it is important that the problems prioritized by the community are not just thought about especially much but are indeed acted on: special resources (including material resources, not just mental capacity) are devoted to addressing them.

So, how does conceptual inflation affect collective attention to problems? Liao and Hansen write that “expressions condemning oppression such as ‘racist’, in their adjectival form, are utterly *un*exceptional gradable adjectives” (Liao & Hansen, 2023, p. 80). There is a sense in which these and other relevant terms are exceptional, however: they have exceptional capacity to guide individuals’ and thereby collectives’ attention to problems. The phenomena recognized as belonging to the corresponding problem categories get prioritized by the community by means of two main mechanisms: those of *emotion* and *commitment*.

Let us look at the emotion mechanism first. Case (2019), as already mentioned, defines conceptual inflation as the exploitation of the emotional impact of the terms. Cikara also remarks that the relevant terms can “trigger bystanders’ anger and engage third-party norm enforcement” (Cikara, 2016, p. 31). The general idea here is that the terms in question evoke emotions (perhaps anger, fear, disgust, or a combination of these) that lead people to mentally foreground the problem and to select it for action. For instance, when a mother reads in the news that students at her daughter’s university complain about rampant sexual harassment, this term’s emotional impact leads the mother to foreground the problem in her mind and might also lead her to take action to address the problem or to call others’ attention to it. For example, she might share the story on social media: stories that are framed in emotionally impactful words are more likely to be shared (Brady et al., 2017). Terms like “racism”, “sexism”, “abuse” and “bullying” likewise plausibly have such a mobilizing emotional impact on many people.

There is ample evidence that emotionally arousing stimuli get mental prioritization—this includes, for example, “potentially fear-relevant material, such as snakes or spiders, pictures of human or animal attack but also erotic pictures and emotional facial expressions” (Kissler et al., 2009, p. 75). The effect has also been observed for emotionally impactful words. For example, identifying ink color takes longer for threat-evoking words like “crash”, “fail”, and “fear” than for neutral words like “clock”, “gate”, and “note”—attention is drawn away from the color-identification task to the emotionally impactful words’ content (McKenna & Sharma, 1995). McKenna and Sharma suggest that “it would make good biological sense to have a response to threat that is rapid and takes precedence over other goals” (ibid., p. 1601). Pratto and John likewise propose that we evaluate stimuli automatically for potential negative effects for our well-being, because an immediate response might be needed, for example, to prevent “loss of life or limb” (Pratto & John, 1991, p. 380). Terms like “sexual harassment” or “racism” may also have the effect of painting the relevant phenomena as something threatening that should take precedent over other goals—thus rapidly drawing our attention to these phenomena.

There are reasons to doubt the reliability of this mechanism, however. Perhaps some of the terms evoke disgust rather than a sense of threat, in some people; and disgust, as Mitchell points out, “typically has an effect on the ongoing modulation of attention which involves *turning away* from the ‘offensive stimuli’” (Mitchell, 2023, p. 77). So, perhaps when some people hear about something “racist”, they react with disgust and are thus motivated to avoid thinking about the disgusting stimulus. The emotional impact of the word would then be counterproductive for mobilizing people to address the problem. Indeed, even a sense of threat might be counterproductive, if it is, more precisely, the sense of being under attack—when the person whose attention we are trying to direct to the problem feels that they are potentially accused of something, and their focus turns to defending themselves. That the term “racism” evokes such a defensive reaction is, in fact, a reoccurring concern. For example, Anderson writes that the term “provokes unproductive, defensive reactions and shuts down urgently needed discussion” (Anderson, 2010, p. 48).^1^ Furthermore, the significance of our initial emotional reaction to the terms is limited by our ability to reflect on and resist the immediate attentional dispositions. We can think about the problem raised to us and find that, on reflection, it does not deserve our attention. That is why it is important to recognize that the power of the terms in question to shape collective attention does not rely solely on the words’ mobilizing emotional effect.

The *commitment mechanism* operates even when the term does not elicit mobilizing emotional reactions. We prioritize problems that are indicated using a certain term (such as “sexual harassment”, “bullying”, “human rights”), when we ourselves are, or our institution or informal group is, committed to addressing problems of the relevant category. For example, the university’s rector might not have a (mobilizing) emotional reaction to the term “sexual harassment”; but the rector is still led to prioritize the problem that is indicated using this term, in mind and in action. This is because the university is publicly committed to addressing sexual harassment promptly and resolutely; and the rector is responsible for ensuring that the university lives up to its commitments. The commitment mechanism also explains why extending the term “addiction” to compulsive gambling motivates research on treating compulsive gambling. Some individual researchers and research groups are committed to a research focus on treating addiction. More generally, addiction is already recognized as an important issue for the discipline of psychology. When compulsive gambling now becomes recognized as an addiction, it thereby becomes something that these individuals and research groups, and the discipline in general, are committed to addressing. Similarly, Cikara points out that “labeling less prototypic prejudice as prejudice may make it easier for institutions—from human resources offices to the justice system—to garner resources and funds to provide better support services to those in need” (Cikara, 2016, p. 31). The commitment mechanism helps to explain how this works: institutions are committed to addressing prejudice; so, they can justify expending resources and funds on these less prototypic cases when these are categorized as prejudice.

An aspect of the commitment is an intention to prioritize certain problems; but the intention is often backed up by public vows and cannot be changed without a backlash. Suppose, for example, that a university is committed to “academic freedom”, and the term’s usage changes so that it becomes easier to indicate infringements on academic freedom. Say, student protests against the content of their professor’s research are now widely considered to infringe on the professor’s academic freedom. A university where such protests take place is then under pressure to prioritize addressing this (alleged) problem, in order to uphold its commitment to academic freedom. For another example: according to Clément, “Beginning in the 1980s … transnational feminist networks successfully appropriated human rights to frame the issue of violence against women, which led to increased mobilisation around the world” (Clément, 2018, p. 157). That mobilization likely drew on activists’ and institutions’ prior commitment to defending human rights, not (merely) the emotional impact of the term “human rights”.

The two mechanisms are not quite independent. Plausibly, institutions are more likely to adopt a public commitment to prioritize problems of emotionally arousing categories like “sexual harassment”—perhaps because there is more public demand for such commitments. On the other hand, individuals are more likely to be emotionally moved by terms in a mobilizing way when they are themselves committed to addressing the corresponding problems, or when they are aware that many other people and institutions are thus committed (so, they are aware that the problem is considered important in the community).

The redistribution of collective attentional resources, now, emerges from individual mental prioritization, which is in turn caused by the emotion and commitment mechanisms. For example, the rector allocates first their own and then the university’s attentional resources to the problems on campus that are categorized as “sexual harassment”, because of the university’s commitment to addressing sexual harassment. Gardiner (2022) provides a helpful characterization of the collective attentional resources that can be thus reallocated:

Funding, space, and time are key attentional resources for most collective agents. But, of course, different collective agents have varied kinds of resources. Attentional resources can include a newspaper’s column inches, an art gallery’s wall space, and a university’s campus layout. Is the library the focal point, for example, or the football stadium? And which departments are relegated to campus peripheries? Accessibility of information and similar abstract features of social infrastructure determine—and can constitute—attention. (Gardiner, 2022, p. 58)

I have argued, then, that when our linguistic practices allow using terms like “sexual harassment” or “human rights” to talk about certain phenomena—say, street harassment or violence against women—this helps to draw individuals’, and thereby the community’s, attention to the phenomena. It is not hard to see how this can lead to debates about how we should respond when it becomes easier to indicate problems that are prioritized through the emotion and commitment mechanisms. Attention, as prioritization, is a limited resource. We cannot prioritize everything: individuals cannot have everything in the front of their minds and communities cannot put special effort into addressing every problem. In James’s words, attention “implies withdrawal from some things in order to deal effectively with others” (James, 2022/1890, p. 404). A collective can prioritize more than an individual, of course. Whereas an individual must withdraw from *x* to deal effectively with *y*, a community can involve groups and individuals that are devoted to *x* alongside those that are devoted to *y.* A collective’s occurrent attention is also not limited to a single human’s working memory. But even a collective cannot prioritize everything of significance.

Some welcome the allocation of more attentional resources to the subtler problems; others worry that this takes some attention away from the most important problems. For example, Haslam worries that the inflating concept of mental disorder could “deflect resources away from more severe conditions” (Haslam, 2016, p. 8). It makes good sense to debate whether we should use “mental disorder” in a broader or narrower way, because the usage of this term influences what the relevant medical and research communities devote resources to. Likewise, it makes good sense to debate whether we should use the term “sexual harassment” in a broader or narrower way, because this influences what behaviors capture people’s attention through spontaneous emotional reaction to the term, as well as what behaviors institutions (such as workplaces or schools) are especially committed to addressing. Those defending a narrower concept of sexual harassment wish to keep the community’s spotlight firmly on the worst cases—perhaps cases that most directly and severely threaten women’s equal participation in work and education. Others want more attention to the effects of the accumulation of smaller indignities. Some worry that the phenomena raised to the status of prioritized problems are not even (slight) problems—that a mountain is made not from a molehill but from nothing at all.

So, disagreements about what should get the prioritization afforded by these mighty words plausibly often reflect political (or more broadly policy-related) disagreements about what is an important problem or not, and whether something is problematic at all. For example, arguing against the inflation of “human rights”, Clément writes: “Framing grievances as human rights places them above the pragmatic considerations of policy-making. It frames the issue in stark terms and, thus, limits any option for compromise” (Clément, 2018, p. 159). But those who want what Clément calls “social justice issues” to be framed as human rights issues think that these issues indeed should be rarely if ever up for compromise—they are that important. Theilen argues that welfare rights are seen as threatening because they “fall outside a (neo)liberal conception of human rights which protects and sustains, or at least does not threaten, the rule of the free market” (Theilen, 2021, p. 847). This is because prioritizing welfare rights on par with “traditional” human rights “would constitute a form of state intervention incompatible with the free-market order” (ibid.). We can now see why our side in the disagreements about the conceptual inflation of problems does not depend only on how pernickety we are about “correct” language. It depends instead on our views about what problems are especially important and what phenomena are problematic in the first place.

The account also helps to make sense of disagreements among those concerned with the role of the relevant terms in theorizing and research rather than in public conversation and policymaking. As I argued above, simply classifying phenomena together does not improve our understanding of the similarities and connections between them (just as classifying them apart does not improve our understanding of the differences). However, since the relevant terms govern researchers’ patterns of attention—via the research community’s commitment to investigating the full scope of the problems of the relevant categories—the wider usage plausibly encourages casting a wider net in investigating the connections and similarities. For example, as Cabezas (2024) argues, a wider notion of racism, which includes institutional racism, encourages investigating the causal connections between individual racist attitudes, institutional practices (like word-of-mouth hiring) with an unintended racial impact, ideologies (like the color-blind ideology) that legitimize these practices, and how all these phenomena together contribute to the overall system of racial oppression. My account—in particular, the commitment mechanism—makes it clearer how the widening of the problem category encourages inquirers’ attention to the wider phenomenon and to the causal connections between the sub-phenomena.^2^ Further, the anti-inflation side may be concerned that focusing on these causal connections and the broader system of oppression distracts the inquirers from honing in on what they regard as the central or fundamental phenomena (such as racist intentions and beliefs). Recognizing and exploring the broader system remains possible, with a narrower usage of “racism”, but is not encouraged to the same extent. The disagreement, then, would be about priorities in inquiry.

While I have presented the attention allocation account as an alternative to a focus on communication and understanding, one might perhaps construe the proposal as another way of fleshing out the idea that communication and understanding are at stake in the disagreements. The anti-inflation side might then be insisting that when a problem category widens and thus comes to include lower-priority problems, our language inhibits us from communicating and understanding the actual comparative attention-worthiness of the sub-problems within the category. And the pro-inflation view might then be that the widening of the problem category precisely allows us to better capture and express the real comparative attention-worthiness of the problems, in thought and communication. In any case, the main disagreement would still ultimately be about whether something is a sufficiently important problem (or a problem at all) and thus deserves to be prioritized by the community in the allocation of its attentional resources.

Note also that this is not an account of why the conceptual inflation of problems has happened. The view is that an *effect* of conceptual inflation is raising new problems to priority status, and that this can be a reason to support the inflation (if one supports treating the newly included phenomena as prioritized problems, generally or in the relevant context) or to oppose it (if one is against the changes in priorities). So, the present account is not in competition with, for example, an account according to which the linguistic changes are explained by the falling prevalence of the problematic phenomena (Levari et al., 2018; Sunstein, 2023).

Suppose, then, that the linguistic changes’ effect on the allocation of collective attentional resources indeed explains why it seems so important to either keep the relevant categories of problems narrow or to broaden them up. How shall we move forward with the debates now that the stakes are clearer?

## Moving forward with the debates

The rational way forward involves identifying and addressing the empirical and normative issues concerning the redistribution of attention through changes in linguistic practices.

The empirical issues concern the details about the mechanisms whereby the changing linguistic practices reallocate collective attention, the further effects of this reallocation, as well as other effects of the linguistic changes. For example, an empirical issue that concerns especially the emotion mechanism, and the commitment mechanism in so far as it operates through personal commitment, is how the problem category’s widening influences the term’s mobilizing emotional impact and people’s personal commitment to addressing the problems under the category. Perhaps extending “racism”, for instance, to a wider range of problems indeed makes us take more seriously issues like structural discrimination. On the other hand, perhaps the term gradually loses its impact, which derived from the term’s association with the most deplorable intentional kinds of racism. Or activists might lower their level of personal commitment to fighting all instances of racism, because of the widening. Considerations in the vicinity have been raised in the discussion on conceptual inflation (Haslam, 2016, p. 14; Case, 2019; Tasioulas, 2021). Dakin, McGrath, Rhee, and Haslam provide some empirical support for the “trivialization hypothesis”: “that exposure to marginal examples of a concept would lead participants to view the harm associated with it as less serious than those exposed to prototypical examples” (Dakin et al., 2023, p. 72).^3^

We should not forget, however, that in addition to emotional impact and personal commitment, there is the more stable institutional commitment mechanism. One might insist, of course, that institutions will also gradually lower their level of public commitment to fighting sexual harassment, for example, in the light of changes in how the term “sexual harassment” is used. But this is surely an empirical question and the answer is not obvious. Furthermore, the terms’ mobilizing emotional impact plausibly derives partly from awareness that many institutions are committed to prioritizing the problems of the relevant categories—that these problems are regarded as important in our society—and not just from their association with especially egregious cases. So, we should expect the emotional impact to remain intact to some extent just because the institutional commitment remains intact.

Another empirical issue is the following. It is sometimes said that the conceptual inflation of problems (injustices, harms, disorders, and so on) contributes to a perception of oneself as a victim. When the categories of problems enlarge, more people see themselves as personally affected. Haslam et al*.*, for example, worry that “[s]eeing oneself as a permanently wounded victim of a trauma may be less conducive to resilience than interpreting the experience in less catastrophic terms” (Haslam et al*.*, 2020, p. 277). Whether conceptual inflation indeed promotes such a sense of helpless victimhood is a non-obvious empirical matter. If conceptual inflation helps to promote previously neglected problems to priority status in the community, then perhaps those affected by the problems instead gain a sense of assurance that their problems are acknowledged as important and in need of addressing.

The central *normative* issue is: Should we encourage the reallocation of collective attention, or resist it? Do the newly prioritized problems deserve that status? A natural suggestion is that the community should devote its limited resources to a problem in accordance with the problem’s importance. For example, Siegel, discussing the ethics of attention in journalism, formulates the “importance principle”: “[M]ake salient information that is important for the public to know about” (Siegel, 2022, p. 237). But how are the anti- and pro-inflation camps to adjudicate whether, for example, street harassment is important enough to be prioritized in the allocation of collective attentional resources (by extending the term “sexual harassment” to it)? Is importance in the eye of the beholder? This seems to be a rather fundamental question about the ethics of collective attention.

The ethics or normativity of attention is a recently flourishing area in philosophy (see Gardiner, 2022; Watzl, 2022; Wu, 2024 for overviews and discussions). Work on the ethics of *collective* attention—the proper allocation of collective attentional resources—is still scarce. Gardiner points out that good collective patterns of attention—say, in a research group—might benefit from individuals’ unhealthy obsessions with narrow subject matters: “A group might exhibit a well-balanced attentional distribution precisely because each group member is differently fixated” (Gardiner, 2022, p. 57). As we have also seen already, some collective attentional resources (such as the design of shared spaces) are importantly different from individual attentional resources (such as working memory). How to even think about the ethics of collective attention is, as of now, still very unclear—in part because it is unclear how to even think about collective attention. But we do need to think about this, to make progress with the disagreements on the conceptual inflation of problems. Here, I will only sketch a view that demystifies the crucial notion—“importance”.

On this view, establishing what deserves prioritization is not a matter of establishing what is timelessly important enough. It is, instead, a matter of negotiating shared goals and distributing the community’s attention to problems accordingly. “Important enough”, on this view, means “important for achieving what currently ought to be our shared goals”. When goals are achieved, new goals should arguably be set, and new problems can then deserve priority status. For example, perhaps now that we have largely achieved our goal of eradicating the worst forms of workplace sexual harassment, we should prioritize the subtler problems in the vicinity and those occurring in different contexts. Accordingly, we should expand our understanding of the term “sexual harassment”, in order to shift our critical eye toward what matters most *now*. Hardimon suggests a similar view about “racism”: “If the word “racism” is to be brought to bear on serious racial ills standing in the way of racial progress *in the present age*, it is essential to allow its application to racial wrongs that fall short of the most severe” (Hardimon, 2019, p. 232; my emphasis).

## Concluding remarks

Changes in linguistic practices are common and often they do not cause serious controversy. But changes in how we use terms like “sexual harassment”, “racism”, “human rights”, or “censorship” inspire debates with political undertones—although the relevant views on policy are sometimes downplayed, and mere interest in preserving language as a common medium for thought and interaction is emphasized instead. These pretensions to neutrality contribute to a focus on whether the conceptual inflation of problems threatens or enhances communication about and understanding of the problems. The effect on communication and understanding, however, is dubious. The flexibility of our language and our own mental capacities allow us to describe problems to others and to arrive at apt perspectives ourselves, regardless of whether certain categories of problems, like racism or sexual harassment, are understood more broadly or narrowly.

Perhaps, then, what the anti- and pro-inflation sides really care about—or should care about—is the effect of conceptual inflation on the community’s allocation of its attentional resources. There are plausible mechanisms whereby problems that can be indicated using the terms in question get prioritized by individuals, institutions, and ultimately the community. The problems come to loom large in the community’s (so-to-speak) mind—they are allocated more of the collective attentional resources—by first looming large in individual minds, in virtue of the interdependent emotion and commitment mechanisms.

This inquiry has been a purposely big-picture one, inevitably occluding details of the controversies about particular concepts. I do not insist that all these controversies must be completely refocused on the effects of conceptual inflation on collective attention, or that all parties to these controversies are already implicitly motivated by conflicting views on this. I do insist that the redistribution of collective attentional resources is an important effect of the conceptual inflation of problems and that this helps to make sense of and constructively redirect many disagreements between the pro- and anti-inflation sides.

## Footnotes

1 A possible response from the pro-inflation side is that our strategy for overcoming race-related injustice need not involve mobilizing everyone in a joint pursuit of justice, including those—usually white people—who react to the term “racism” defensively. Cikara, for example, suggests that we could prioritize mobilizing the victims of injustice—turning their anxiety into certainty and anger—and that “some degree of conflict is required for social progress” (Cikara, 2016, p. 30). See also Hardimon, 2019.

2 I think this is a plausible way of fleshing out Cabezas’s own suggestion that “applying the label ‘racist’ to the institution encourages us to adopt the macroscopic view of oppression” (Cabezas, 2024, p. 211).

3 On the other hand, Hansen and Liao’s (forthcoming) results from a study using a different methodology (comparing how different age cohorts interpret the term) suggest that the felt intensity of the term “racist” has not diminished.
